# Our Paris Letter

**Published:** 1891-10

**Authors:** T. M. McIntosh

**Affiliations:** Thomasville, Ga.


### Correspondence.

### OUR PARIS LETTER.

PARIS, FRANCE, June, 1891.

EDITORS SOUTHERN MEDICAL RECORD :

I left New York on April 30th for a stay of some months in the German and other hospitals with the purpose of studying the men and the methods of the European countries. A few notes gathered by the wayside may be of some interest to some of your readers, even should they not obtain many new or valuable ideas therefrom.

A few days were spent in London, where one has much difficulty, by reason of the great distances of the city and the widely scattered sites of the various hospitals, to do much work. The reception of visiting physicians, as a rule (not at every place) it seemed to me, was not very cordial. But I had the satisfaction of seeing many interesting cases, the greatest of which was an operation, by Sir Joseph Lister, for fracture of the patilla, y wiring the fragments of bone together; and at the same time a very interesting lecture upon the details of the operation, the most important of which was, that all periosteal and other tissue which would fall between the fractured surfaces of the bones should be carefully cut away, so that bone surfaces should be in contact, as only bone cells could generate new bone cells and tissue.

In Edinboro, Scotland, at the Royal Infirmary, one can not only see a great deal of interesting work, but be sure of a cordial reception.

Mr. Annandoli's surgery is skillful and bold, as by every one known. For the study of medicine (internal medicine particularly) Edinboro is better in my judgment than London. The students hire an elegant clubhouse where they can dine cheaply, or the contrary, if they wish, play billiards, read, smoke and be otherwise sociable, and pleasantly entertained. But, I began to write only of Paris. I have been here nine days, and during that short time I have been the recipient of many courtesies from the professional medical men. Everyone should, if possible, visit Pasteur's Institute, and even those who do not believe in the existence of hydrophobia, would certainly be profoundly impressed with the earnestness of these workers here, in this department of experimental and prophylactic medicine. Let no one imagine, however, that this Institute is devoted only, or even chiefly, to the experimental study and the treatment of rabies, for it is in fact a superbly equipped Institute for study in all departments of microscopy, and of all contagious and innocuable diseases. There is a large lecture room, a room where Bouillon, Agar and Gelatine (the culture fluids) are prepared. One man does only this.

There is a room used only for trepanning. Cows, horses, chickens, pigs, cats, dogs, rabbits, mice, birds are kept here in great numbers, each in its special place, for experimental purposes. The students in the laboratory buy these animals at half price, when they wish one for experiment, and the Institute feeds them free of charge. Dr. Roux is making investigations as to the immunity of anthrox by innoculations ; intestinal diseases of children, tuberculosis, diptheria and glanders.

Prof. Metschinkoff, who is famous for his studies of the leucocyte or wandering cell, and promulgated the Phagocyte theory, is still making observations on the functions of this little health sentinel of the human blood, which is ever alert and ready to see, and seize upon and devour every invading microbe which may enter the blood from without, through wounds, the respiratory or digestive organs, or form within by auto-infection. In healthy organism, if the specific attacking microbe is not in too great number, they are devoured by the defending Phagocyte and the organism remains sound. Per contra, in the enfeebled constitution the ground is too favorable for the microbe of disease, and their multiplication is so rapid and in such quantities, that the Phagocyte can not destroy them, and disease is the result.

This is the theory. The facts are, that abnormal cells are found in great numbers in the blood of diseased organisms, and diseased organs or localities, and not in healthy blood or localities ; and that the Phagocyte is found in great numbers in healthy blood organs, and in less numbers where there is local or general diseases. These observations may have an important bearing on surgical pathology; and it seems to me to throw light upon the correct principle of wound treatment, the aim of which should be, not the destruction of extraneous substances and ferments which get into the wound by the use of antiseptics, which would impair the resisting or healing power of tissue as well as to destroy, perhaps, the infecting microbe, but by placing the wounded tissues in a condition as near to that which existed normally before operation or injury, as is possible, so that the development of the Phagocyte is favored and his vantage ground of attack and defense is increased, and that of his enemy diminished. This is best brought about by an absolute, as possible, absence of contact or entrance *into* the wound of all substances or things not a natural part of the tissues, which condition is not favored by the introduction of antiseptics into the wound, but by an absolute asepsis, (sterilization) without antiseptics. And this is done by an absolute cleanliness of sponges, instruments, sutures, gauze and every other thing that can possibly come in contact with the wound, by subjecting all of these things to a steam of hot water, temperature of 212 degrees, for one-half to one hour; and a thorough cleansing of the part to be operated on by the use of soap and warm sterilized water. And to properly prepare the above named utensils of surgery in this manner requires more care than to do so by antiseptics, but is more conducive to the rapid and proper healing of the wound. Metschinkoff is yet continuing his observations on the behavior of the Phagocyte, as well as studies in zoology and embryology.

Dr. Duchaux is the chemist of the Institute. I had the pleasure of an introduction to Pasteur himself, who is old, and quite feeble from a recent paralysis from cerebral embolism. The injections for the prevention of rabies takes place every day, and one sees patients from every part of the world, even the Arab and the Turk. There were sixteen new cases, in which I saw the first treatment. A piece of the spinal cord of a rabbit dead of the virus "fixed" by repeated inoculations, is put in sol. caustic potash, and then dried for sixteen days. One-half centimeter of this cord is put in three centimeters of Bouillon, and this is injected into the abdominal wall.

Solutions in Bouillon are made from this cord dried from three to sixteen days, and the treatment is begun with sixteen day dried, the next day the fifteen day dried and down to the third day dried, cord solution.

Every effort is made to prove the existence of rabies in the patients treated. Fifty per cent. of the cases admitted bring specimens with them, 80 per cent. of which are "proven" to be rabies. A little over 15000 cases have been treated, five of which have died, which is an astonishingly good result. The verity of these figures have been attacked, and even the existence of hydrophobia. On this point your correspondent can have no opinion. All who are in the Institute believe in its existence and cure by Pastuer's method. Dr. J. J. Kinyoun, of the Marine Hospital service, and formerly of North Carolina, enabled me, by his great courtesy, to obtain this information. He has been here several months, and previously in Koch's laboratory in Berlin, studying bacteriology, under the direction of the Marine Hospital service, in order to prepare himself for the continuance of this work in Washington, where it is proposed to greatly increase the present facilities for, and widen the scope of scientific laboratory work. This is, as yet, let us hope, only a beginning of what our Government will do in the way of support of men and institutions for original scientific work. Germany, France and England each have great universities, supported by the Government, and as a consequence are all educational and scientific centres, where those interested in any special study or line of original work, have ample money and material at their command.

An afternoon spent in the clinic of Apostoli, the great advocate of the treatment of uterine diseases by electricity, can not fail to be of great interest and much information. He is a man about forty-five years old, five feet ten inches high, 160 pounds in weight, with a face of pronounced features, earnest and expressive when speaking, which he does in a rapid and attractive manner. The whole appearance of the man indicates strength, mental and physical. His clinic is on the second floor of a dirty old house, with an uninviting entrance from a narrow and unattractive street. But all within is clean, and you are made to feel at once at home by a very courteous welcome. The man's earnestness is impressive, and if a large clinic is an evidence of success in treating disease, he is markedly successful.

While one must always resort to surgical treatment, very often, in many cases of uterine disease, which can not be relieved or cured, or the life of the patient saved, except by surgical resources, I am fully convinced that there are many women now submitted to severe surgical operations, that could be treated with as good results by Apostoli's method, and without danger to life; and I refer particularly to interstitial uterine fribroma and myoma which are dangerous to life and health from the free and often protracted hemorrhage which they cause, to relieve which a removal of the tumor, an hysterectomy or an Oophorectomy is the usual resource. Even with the present wonderful success in these operations, each with a mortality of only about 5 per cent., the almost certain prompt control of the hemorrhage, or its diminution within the bounds of health, the frequent relief of pain, and often a diminution of the size of the tumor, obtained by Apostoli's treatment, should make a surgeon hesitate before resorting to a formidable operation, where other, safer, and sometimes just as good means are at his command. If under the Apostoli's method an exhausting hemorrhage will sometimes continue and a resort to an operation is required, it is also a fact that an Oophorectomy does not always relieve the hemorrhage due to interstitial uterine tumors. Apostoli should first be tried, and then, if of no value, surgical treatment is demanded. Such a distinguished and successful surgeon as Mr. Kieth has almost entirely abandoned the surgical treatment of all uterine tumors, except the cystic extraperitoneal, for the method of Apostoli. Also, Apostoli uses his method in almost all of the diseases of the uterus and adnexa. I have notes of a few cases treated without selection, just as the turn for treatment of each patient came; suppurative salpingitis, interstitial Fibroid, endo-porometritis with hemorrhage, prolapsus and paramitritis, ovaro-salpingitis from gonorrhœa, and severe uterine neuralgia. These cases had been under treatment from one month to one year. One case, a woman aged forty-seven, which he had treated and published as cured of parametritis, was examined for the first time in seven years and found only some post-uterine thickening. She had no pain. Of course Apostoli, like every ardent specialist, treats many cases with his method which would, perhaps, do better otherwise handled, and sometimes claims for his remedy that which nature has done.

But perhaps the most interesting hours that I had in Paris were those spent in the clinics of the public and private hospitals of the great Pion, the originator of the Hæmostacic in operation. I was in Paris some days, and the mornings of five of those, even with Dr. Pion, by personal invitation. For these courtesies, aside from the natural politeness and courtesy of all the French physicians, I am under obligations to our distinguished Georgian, Dr. Robert Battey, whose name and whose fame is familiar to all of Europe. Pion is a large, handsome man, inclined to rotundity, with a strong large face, a rather heavy chin and mouth, which is balanced by a high and very intellectual forehead. In his clinics before the class he operates in a full dress suit, but in his private hospitals he dons the regulation white apron of the operating room.

I will mention four of the most interesting operations, because they illustrate better his peculiar method of arresting and controlling hemorrhage.

The first was a vaginal hysterectomy for carcinoma, which, with the woman in the usunl position on the back and the usual specula and retractors in the vagina, he began by making two deep lateral cuts on each side of the uterine cervix, and then grasping the tissues through these cuts with curved hœmostatic forceps, and cutting again the tissues between the uterus and the grasp of the forceps, never letting the incision go beyond the point of the forceps, thus always with a forceps grasping tissue in advance of the incision, so that the hemorrhage would be controlled. In this manner, after nearly an hour, the operation was complete, and the woman had the vagina full of forceps projecting therefrom, and left therein still grasping the tissues that would otherwise bleed. The vagina was packed with a few sponges, filled with iodoform, otherwise the dressing was as usual. In from twelve to twenty-four hours the forceps were removed, and the treatment otherwise as usual. The next operation was the removal of a large intra-mural unterine fibroid with the forceps only as a hæmostatic means, as in the hysterectomy. These were in the hospital for women. For the next day I had an invitation to see at another hospital an operation for ligature of the thyroid artery. I went. The disease, an anuerism. It was cut down upon, fully exposed, a forceps was applied upon either side of the aneurism. Pion arose from his seat with an exclamation of satisfaction, laid aside his knife, paused a moment as if to rest, turned away from his patient to get a ligature I thought, but to my surprise, his assistants began to dress the wound and he to converse. (He speaks some English.) Then forceps were allowed to remain for forty-eight hours, the wound being dressed in the usual aseptic manner, the forceps being completely covered with the gauze and absorbent cotton. The next day he amputated at the hip joint, and grasped the femoral artery as in the other cases, with only forceps, which were removed in seventy-two hours. In all of the operations which I saw him make, not a ligature did he use, or a piece of silk or catgut, except in approximating the skin flaps. He never uses the ligature or suture in controlling hemorrhage. For the vessels of different sizes he allows the forceps to remain different periods of time. The radial artery twelve hours, the axillary forty-eight hours, the femoral seventy-two hours. While, perhaps, but few surgeons except Pion will discard the silk and catgut ligature for the forceps, his method can not fail to be of much interest; and only confirms in practice the teaching of the pathologist as to the length of time required for the promotion of an arterial thrombus, firm enough to prevent all bleeding. In the first use of the ligature of arteries, it was only thought necessary to merely approximate the inner walls, as the mere *stasis* of the blood was thought to be all that was required for the proper promotion of thrombus, and that a firm binding of the artery would deprive the vessel itself, below the point of ligature, of nutrition. In the light of the present knowledge of the power of the endothelium to keep blood in the fluid and healthy condition, and the effects that injury or disease of this sort has upon the promotion of thrombus, the practicability of Pion's method and the correctness of his pathology is demonstrated, for it is quite evident that the injury produced by the grasp of a forceps is greater in area than that of the ligature (as applied at the present time, cutting the two internal arterial coats) and therefore a more firmly adherent thrombus is found, because the area of injured endothelium is greater, and it is at the injured parts that a thrombus is quickly and first deposited and to which it soonest adheres, and where its vascular connections with the nutritive arterial vessels first takes place.

Without expressing a personal preference, as to the relative value of silk or catgut in the ligature of arteries, it would seem that this method of Pion would do away with all fear that catgut would be absorbed before a firm thrombus had been formed, which takes place according to experiments of pathologists, in from two to four days, a time too short for the catgut to be absorbed, and in those cases in which hemorrhage takes place after this use of catgut, the cause would seem to be an imperfect knot and its slipping, or if occurring some days after the operation, the result of a septic and suppurative state of the wound, which prevents a rapid and firm thrombus formation, or produces a breaking down of one already formed.

The average surgeon will no doubt find silk and catgut better than the forceps as a hæmostatic means, but one may have occasion to find it very comfortable, useful too, to know that a forceps can be left for a few days in a wound, holding a large artery, or in the abdominal cavity, holding the pedicle of an ovarian tumor. I go hence to Germany, where I remain 'till December, and perhaps may find something to write of again.

T. M. McINTOSH.

Thomasville, Ga.

